# The Profession of British Nurses: Plain Words on Registration.—IV

**Published:** 1917-04-21

**Authors:** 


### THE MATRONS' AND SISTERS' DEPARTMENT.

### THE PROFESSION OF BRITISH NURSES.

### Plain Words on Registration.—IV.*

WHAT A STATE REGISTER MIGHT ACCOMPLISH FOR NURSES.

There are three professions, analogous in several respects to that of nursing, one of which enjoys a State Register. The others have never sought after such a possession. These are accounting, engineering, and teaching. It would be quite impossible for the State, were it deemed desirable, to set limits to these employments. The young person who keeps the simple accounts of a huckster is, in her way, an accountant. The man who shouts aloud in the racing ring is a Turf accountant, while an accountant of a different order occupies a high place in the Bank of England. Precisely the same condition of affairs prevails in the engineering world. The driver of a traction engine, the fitter who jobs about the machine-room of a factory, the mechanic who fixes an insulator on a telegraph-pole, the designer of a battleship, or of a great railway system, or of a gigantic bridge—all of them are engineers. As for teaching, it permeates every sphere of human activity. The ill-paid and hard-worked mother's help finds teaching a specific part of her duty. There are teachers of music and of art, primary and secondary school teachers, university professors, teachers of science, of cookery, of needlework, of woodwork, of horsemanship, of farming, of poultry-keeping. Eminent physicians and surgeons spend a portion of their time teaching medical students and nurses.

TEACHERS' STATE REGISTER.

The only one of these professions which possesses a State Register is that of teaching, which, indeed, probably corresponds more closely with nursing than either of the others. One outstanding feature common to both is the inadequacy of the remuneration taking account of their necessary attainments. Every competent teacher must be a person possessing expert knowledge, and every class of teaching, with a few shining exceptions, is notoriously badly paid. When they sought for a Register, many teachers had a vague belief that in some way this would limit the market. But it was a vain hope. A responsible and authoritative view of its potentiality was that expressed by the Right Hon. A. H. D. Acland, who, as chairman of the Teachers' Registration Council, expressed a hope that what would follow the granting of a State Register would be "the larger and more general conception of the unification of the teaching profession."

"These words," observed the Chairman, "express an aim which has formed the background of the Council's work. Great as is the diversity of teaching work, and of professional aspirations represented in the Council, its efforts to frame the conditions of registration have been made in the unanimous and sincere belief that the solidarity of the entire teaching profession is worth striving for, not only in the interests of teachers as a body, but also in the interests of education, and of the whole community." In this sentence is summed up the aim of the Council in seeking a register, and of the State in granting one.

May we suggest that the nursing profession sh uld adapt and adopt Mr. Acland's words as a text? It is quite as futile to endeavour to set a limit to nursing as to the other professions or occupations which we have named, and the march of progress continually tends to increase the difficulty. Home nursing is actually being taught to school children as an essential part of elementary education, and the difference between the most highly trained and accomplished nurse and the schoolgirl who wins a certificate in Home Nursing, or between the hucksters' clerk and the Bank of England accountant is merely of degree. Hence it can never happen that the State by granting a Register to nurses can by any possibility confer a monopoly of employment on their profession.

A NURSES' REGISTER.

What a Register might accomplish, first of all, is to render illegal the appointment of any save registered persons to public appointments, as in the case of the medical profession. But at present this principle is, almost without exception, recognised, the word "trained" being substituted for "registered." It is desirable to make this a legal enactment, but, seeing that no abuse at present exists, no appreciable benefit would accrue to the nursing profession. It cannot, indeed, be too forcibly iterated that a State Register would scarcely, if at all, affect nurses' employment, and that, therefore, the economic troubles of the profession, which we hope to discuss in a future issue, are not caused by the lack of a Register. Secondly, a substantial benefit that might accrue to nurses as the result of registration legislation is the bringing about of what Mr. Acland, referring to teachers, described as "the solidarity of the entire profession." This, be it observed, is not a necessary consequence. It has not occurred so far in the teaching profession, and it cannot take place in the nursing profession unless a broad view is taken of the situation. The narrower the limits of a Register, the less its potentiality. We do not desire to quote such platitudes as "Union is Strength," but, strange as it may appear, many would-be leaders of nurses preach from the housetops that only an extremely select

* For previous articles see March 31, p. 521, April 7, p. 15, and April 14, p. 35.

Register can be of service to nurses, which is an obvious fallacy. Perhaps the finest model for the profession is the British Army—invincible, triumphant—not English, Scotch, or Irish, but comprising the sons of the Motherland from the ends of the earth and wherever the flag waves, and recognising the necessity for all the talents from Field-Marshal to drummer-boy. On such lines as these, what a noble army might be formed from the Profession of British Nurses!

